# Definition of an automated Content-Based Image Retrieval (CBIR) system for the comparison of dermoscopic images of pigmented skin lesions

**DOI:** 10.1186/1475-925X-8-18

**Published:** 2009-08-16

**Authors:** Alfonso Baldi, Raffaele Murace, Emanuele Dragonetti, Mario Manganaro, Oscar Guerra, Stefano Bizzi, Luca Galli

**Affiliations:** 1 Department of Biochemistry, Section of Pathology, Second University of Naples, Naples, Italy; 2 Futura-onlus, Rome, Italy; 3 ACS, Advanced Computer Systems, Rome, Italy

## Abstract

**Background:**

New generations of image-based diagnostic machines are based on digital technologies for data acquisition; consequently, the diffusion of digital archiving systems for diagnostic exams preservation and cataloguing is rapidly increasing. To overcome the limits of current state of art text-based access methods, we have developed a novel content-based search engine for dermoscopic images to support clinical decision making.

**Methods:**

To this end, we have enrolled, from 2004 to 2008, 3415 caucasian patients and collected 24804 dermoscopic images corresponding to 20491 pigmented lesions with known pathology. The images were acquired with a well defined dermoscopy system and stored to disk in 24-bit per pixel TIFF format using interactive software developed in C++, in order to create a digital archive.

**Results:**

The analysis system of the images consists in the extraction of the low-level representative features which permits the retrieval of similar images in terms of colour and texture from the archive, by using a hierarchical multi-scale computation of the Bhattacharyya distance of all the database images representation with respect to the representation of user submitted (query).

**Conclusion:**

The system is able to locate, retrieve and display dermoscopic images similar in appearance to one that is given as a query, using a set of primitive features not related to any specific diagnostic method able to visually characterize the image. Similar search engine could find possible usage in all sectors of diagnostic imaging, or digital signals, which could be supported by the information available in medical archives.

## Background

Melanoma and non-melanoma skin cancers currently constitute one of the most common malignancies in the caucasian population, and the worldwide incidence and mortality rates are continuously increasing [1]. In particular melanoma incidence has increased more than any other cancer, reaching currently 18 new cases per 100.000 population per year in the United States [2]. Because advanced skin cancers remain incurable, early detection and surgical excision currently is the only approach to reduce mortality.

The traditional screening tests require a skin naked-eye examination by an experienced clinician. One of the most widely used method for evaluating with naked-eye pigmented skin lesions (PSLs) is the ABCD rule [3]. However, this system may fail to detect many difficult or borderline PSLs which are small or/and regular in shape or color.

Dermoscopy (dermatoscopy, epiluminescence microscopy, incident light microscopy, skin surface microscopy) is a non-invasive diagnostic technique for the in vivo observation of PSLs, allowing a better visualization of surface and subsurface structures (epidermis until the papillary dermis). This diagnostic tool permits the recognition of morphologic structures not visible by the naked eye, thus opening a new dimension in the analysis of the clinical morphologic features of PSLs. Several studies [3] have shown that this method may improve diagnostic sensitivity by 20-30% compared with clinical diagnosis by naked eye. However, due to the complexity of patters and their interpretation, the results of dermoscopic examination have limitations especially for the inexperienced and they are effective only if the users are trained formally. In order to reduce the learning-curve of non-expert clinicians and to mitigate problems inherent in the reliability and reproducibility of the diagnostic criteria used in pattern analysis, several indicative methods based on diagnostic algorithms have been introduced in the last few years. The ABCD rule, the 3-point checklist, the 7-point checklist, the Menzies's method and the CASH algorithm are the most relevant ones [3-5].

Recently, numerous systems designed to provide computer-aided analysis of digital images obtained by dermoscopy have been reported in literature. The aim of these systems is to transfer the ABCD attributes, as well as other characteristics based on texture, into automatically computed quantities and use these parameters in order classify the PSLs [6]. The proposed computer-assisted methods differ from each other depending on the set of features extracted on the digitized dermoscopic images and on the feature selection and classification methods used. Despite multiple publications which assess the improved diagnostic accuracy of these instruments compared with that of clinicians, the effectiveness of these systems depends largely on the dataset used [7,8]. Several studies have shown that currently, the proposed image analysis systems are able to identify correctly the clinical obvious melanomas, but they have limited capabilities to discriminate between border line lesions and early malignant melanoma [8]. Therefore, so far, these computer-assisted diagnostic imaging tools provide little benefit for the experienced clinician [6]. Melanomas and certain forms of benign skin tumours differ slightly in their physical characteristics: hitherto no single discriminating parameter (or set of parameters) has been discovered. An experienced clinician bases her/his decision on experience, as well as on complex inferences and extensive knowledge which is very difficult to mimic in a mathematical algorithm [9].

In this manuscript, we do not propose another PSL classification system, but a content-based image retrieval system for dermoscopic images as a diagnostic aid to the clinicians for skin cancer recognition. We argue that this kind of system, which is effective in retrieving PSL images with known pathology visually similar to the image under evaluation, may provide an intuitive and effective support to both inexperienced and experienced clinicians which can improve their diagnostic accuracy. Despite the large proliferation of CBIR scientific paper in literature, only very few papers deal with the CBIR systems applied to dermocopic images. To the best of our knowledge there is only one proposed by Rahman et al, and typically the proposed CBIR systems are validated using small PSL data set (~400 images) [10].

Computerized methods enable storing large set of images together with diagnostic information for further investigations or creation of new methods of diagnosis. The large diffusion of digital archiving systems of dermoscopic images in medical institutions results in an increasing research interest to develop new approaches for more efficient exploitation of these huge amounts of clinical data. Recently, several research efforts have been focussed on using Web and Semantic Web technologies in medical image databases, in order to better reuse and share all the information they contain as well as to overcome the current state of art text-based access methods in medical information retrieval [3,11].

## Methods

### Selection of PSL for evaluation

From January 2004 to September 2008, 3415 patients were examined independently, as part of routine skin cancer screening, by three of the authors (R.M., E.D., A.B.). All patients were Caucasian. The examination included clinical inspection and digital dermoscopy of PSLs. These were classified as either benign, or as suspicious of being malignant, following internationally recognized dermoscopic parameters [12-18]. PSLs considered suspicious by the clinicians were excised and subjected to examination by two independent pathologists. For ethical reasons, clearly benign lesions were not excised. They were also included in the database, since specific aim of the project was to develop a system suitable for the whole range of lesions, not only for the borderline or malignant cases.

The final number of selected PSL cases was 20491. Diagnostic categories of the 24804 images are indicated in Table 1. Clinical data, obtained from each patient, included: age, sex, skin photo type, location, diameter, and duration of the lesion. The locations of the PSLs included in the study were as follows: head-neck 458 images, acral sites 2380 images, nails 7 images, mucosa 107 images, rest of the body 21852 images.

**Table 1:** Diagnostic categories of 20491 PSLs included in the study

*Diagnosis*	*N° of cases*	*N° of images*
Melanoma	94	288
Atypical nevi	1785	2677
Benign nevi	18160	21155
Congenital nevi	189	331
Blue nevi	60	105
Combined nevi	18	21
Ungueal nevi	5	7
Lentigo	45	53
Melanosis	80	107
Basal cell carcinomas	55	60
*Total*	20491	24804

### Dermoscopic image acquisition and data selection

The three clinicians performed dermoscopy using exactly the same apparatus. In particular, dermoscopy was carried out with a digital reflex camera with a colour 12.3 megapixels (4288 × 2848) 14 bits CMOS sensor of dimension 23.6 × 15.8 mm. The reflex camera employs a LiveView technology, which allows the users to shoot at high or low angles with the live preview on the LCD monitor. A wireless transmitter system enables complete remote camera control and fast image transfer (up to 54 Mbits/sec) to a computer. Constant illumination is provided by a 24 white light-emitting diodes (LEDs) in a ring arrangement. A calibration device made of colour calibration ramps is inserted within a slide which is targeted by the acquisition system illumination. Absolute radiometric correction of every single image acquired is, therefore, performed by making the calibration colour scale uniform across the entire archive. The maximum visual field of view for dermoscopic images was 14 mm in vertical dimension corresponding to an effective pixel size on the skin of about 5 micrometers.

### Images retrieving

**Figure 1:**
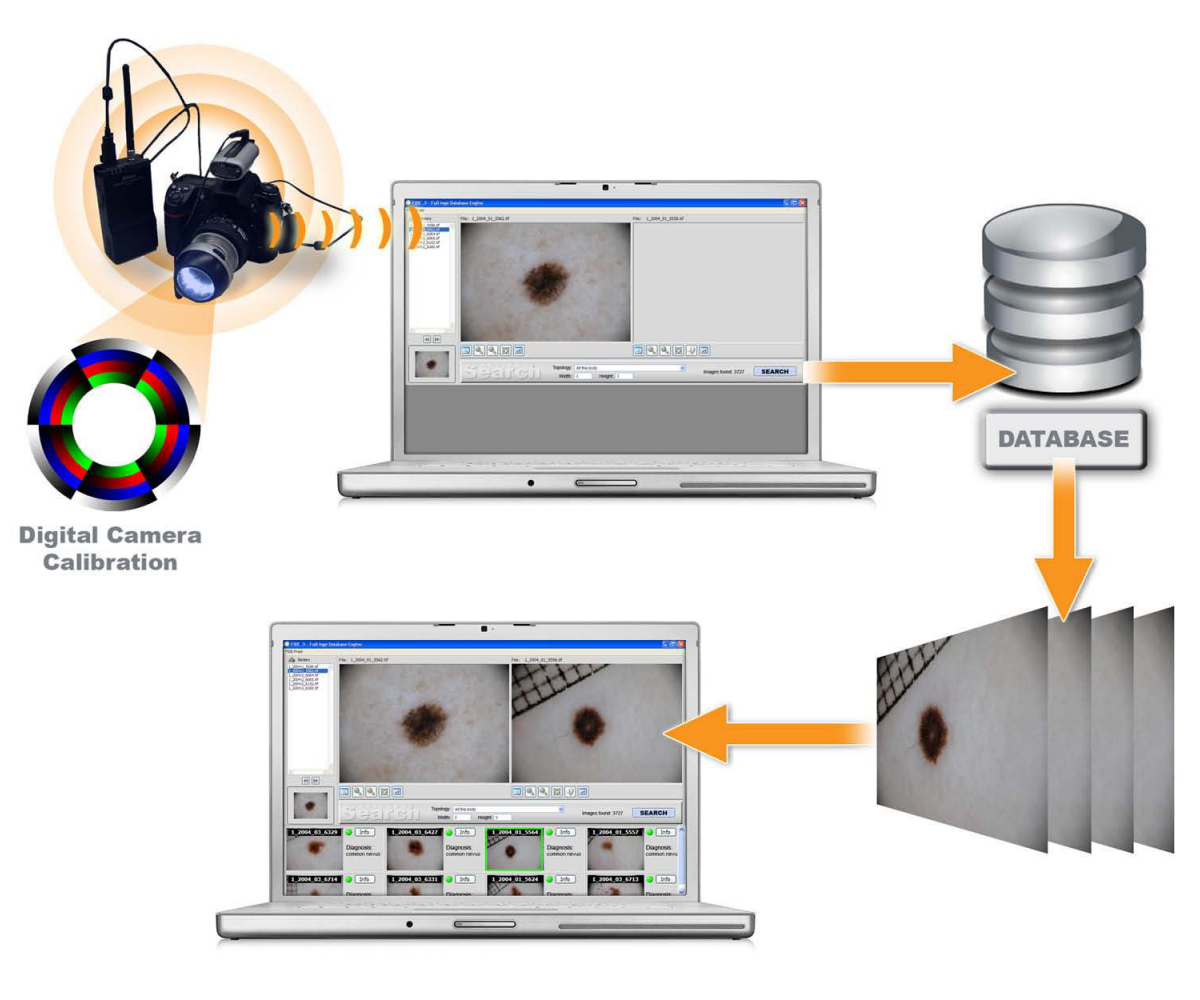
**General scheme of the system**. Dermoscopic image acquisition and search engine.

The PSL images are acquired with a well defined dermoscopy system (Figure 1) and stored to disk in 24-bit per pixel TIFF format using interactive software developed in C++. Successively they are ingested into the analysis system. The ingestion consists in the extraction of the low-level representative features which permits the retrieval of similar images in terms of colour and texture from the archive.

Colour characterization is performed by computing the scatter-plot of Red channel vs. Green channel, the Green channel vs. Blue channel and the Blue channel vs. Red channel. The proposed colour description is not invariant to illumination changes. However, it is fully adequate in conjunction with archives of absolute calibrated dermoscopic images acquired with dermatoscopy systems similar to the proposed one described in the material and methods section.

Texture assessment module is based on the colour ratio (Red/Green, Green/Blue and Blue/Red) co-occurrence matrix (CRCM) and a local image variance. The CRCM is defined as joint probability occurrence of colour ratio pixel pairs with a definite spatial relationship specified by a certain displacement and angle. The CRCM is preferred to the more used grey level co-occurrence matrix (GLCM) [19], because several experiments revealed it is more informative for skin lesion images: GLCM and CRCM distributions are both heavily picked along the its principal axis but GLCM presents sensibly less dispersion along the other directions than the CRCM. The pixels with very low-value grey levels provide unstable band ratio information. Therefore, the low-intensity pixels are masked when computing the CRCM. In order to obtain rotation invariant features, the normalized CRCM is computed for each of four orientations: 0°, 45°, 90°, 135°. Multi-resolution analysis is performed using CRCMs at several displacement steps: 5, 15, 30 and 60 pixels. The colour ratio is uniformly quantized into 255 different values, and a separate multi-scale CRCM is computed for each colour ratio couple. The local image variance is computed for each colour band using a moving window with size of 11 × 11 pixels, it is normalized to the (0,1) interval and uniformly quantized into 255 different values. The corresponding scatter-plot with respect to the image pixel radiometric value is computed, and properly normalized in order to make this feature independent of the skin lesion image size.

### Archive access method

For interactive CBIR systems the archive access method is very important issue in order to keep the response time at few seconds even with large archives of thousand of images. This is particular true in the proposed CBIR system, where multiple scatter-plots and co-occurrence matrices are used in order to have an accurate representation of the skin lesion images in the feature space: three colour scatter-plots (one for each band couple), three local variance scatter-plots (one for each band) and three colour ratio (Red/Green, Green/Blue and Blue/Red) co-occurrence matrix (CRCM) containing all the four displacement steps (5, 15, 30 and 60 pixels). Each skin lesion image is therefore represented by 255 × 255 × 3 × 3 variables. A straightforward computation of the Bhattacharyya distance of all the database images representation with respect to the representation of user submitted (query) image may require several hours of CPU time for an archive of 25000 images.

A three steps hierarchical multi-scale approach is proposed in order to radically reduce the system response time. First the complete ranking of the database images is performed using scalar parameters extracted from the feature space image representation. Only the top *n *ranked images are picked up for the second selection step, where the image ranking is carried out using reduced resolution scatter-plots/co-occurrence matrices. The reduced resolution image representation is composed by 16 × 16 × 3 × 3 variables. The final image selection is performed by means of the top *m *ranked images using the complete image representation.

The experimental procedure to obtain m and n parameters was the following:

1. set n = m = 10000

2. for each query image belonging to a subset PSL image archive compute the corresponding first 100 retrieval images

3. set the archive access parameter (n-1, m-1) and repeat step 2

4. compute the difference between the retrieved images computed with (n, m) and (n-1, m-1) for each query image. The difference is computed summing the score values relative to the retrieved images which are different. The score is defined by the inverse of the ranking position. For instance, in the case the retrieved images placed at the 10th position and 20^th ^position are different the relative difference is 0.1 + 0.05 = 0.15.

5. if the mean difference is below the threshold T = 0.1 reduce (n, m) = (n-1, m-1) and repeat steps 2-5, otherwise continue

6. keep n value fixed and reduce m value m = m-1

7. for each query image belonging to the subset PSL image archive compute the corresponding first 100 retrieval images.

8. compute the difference between the retrieved images computed with m and m-1 for each query image as done at step 4

9. if the mean difference is below the threshold T = 0.1 reduce m = m-1 and repeat steps 7-9, otherwise continue.

The scalar parameters extracted are: the average colour lesion values in the RGB space, the average local variance value for each RGB band and mean and variance Haralick parameters [20] extracted from each CRCM (five different displacements and three different band ratio couple). They form a thirty-six dimensional feature vector . The distance metric used is the weighed Euclidian distance:



where the weighting factors are those used at high resolution feature space and reported.

The *n *and *m *values are chosen experimentally in such a way as to not alter significantly the response accuracy. The results reported in Figure 1 are achieved using *n *= 2000 and *m *= 100. Using an Intel-based Pentium Core 2 Duo 1.6 GHz with 2 GB of memory, the related average overall response time for an archive of 25000 images is of 5 sec. Increasing the archive dimension and keeping *n *and *m *constant, only the access time relative to the first step linearly rises with the archive image number, which is less than 1/20^th ^of the overall response time.

## Results

We have defined a novel content-based image retrieval (CBIR) system for dermoscopic images able to recognize analogies between images or image fragments as a diagnostic support to the clinicians for skin cancer recognition. Concerning image indexing, and using the nomenclature proposed by Datta et al [21], the PSLs images are described using global color features and local texture features (colour ratio co-occurrence matrix (CRCM) and a local image variance). The image signatures used is the "summary of local feature vector" as discrete probability density distribution (two dimensional scatter plot), the distance ("dissimilarity measures") is the Bhattacharyya distance metric.

**Figure 2:**
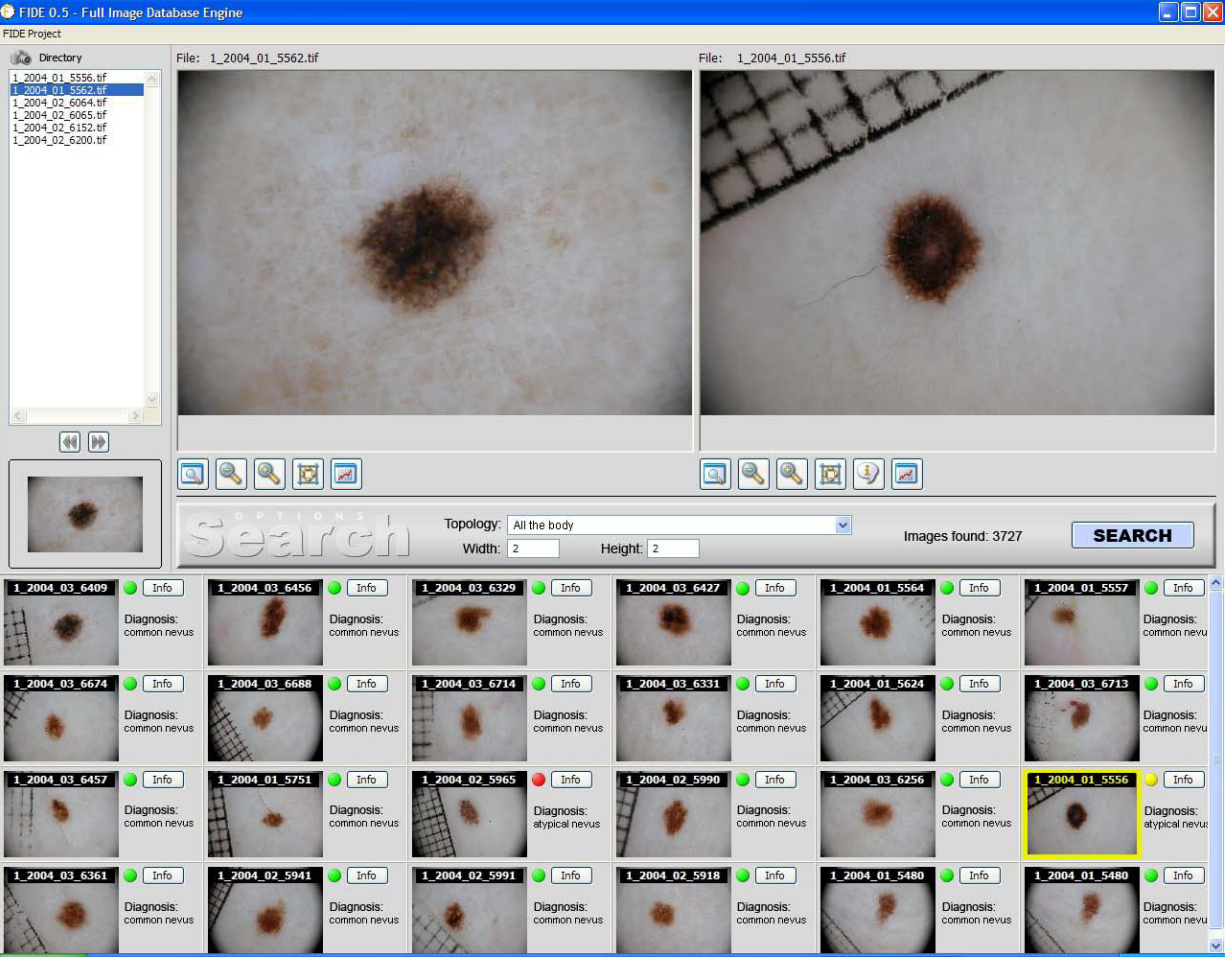
**The search engine system user interface**. The upper left-hand corner shows the digitized PSL query image. The bottom panels show the first 24 images from the similarity ranking list that have been retrieved from the PSL database together with the relative diagnostic class (green bullet: common nevus, red/yellow bullet: atypical nevus, black bullet: melanoma). A more detailed diagnostic report can be visualized by clicking the "info" button. The upper right-hand corner shows the magnified image of the user-selected retrieved PSL.

The aim is to aid decision making by locating, retrieving and displaying relevant past cases along with diagnostic reports. One of the most challenging aspects in this domain is to extract local lesion specific image features and define the image similarity model. An image is usually modelled as a collection of low-level image features (colour and texture). Image similarity computation entails the use of different similarity measures on the extracted image features. The synthesis of these specific criteria yields the overall image similarity measure to the query image, allowing the complete ranking of the database images with respect to the user submitted (query) image. In the proposed system, the first n-images from the similarity ranking list are selected and displayed to the user together with the relative diagnostic reports. A general scheme of the proposed system is reported in Figure 2.

The characteristics of the PSLs collected for the study are depicted in Table 1. The PSL images are acquired with a well defined dermoscopy system (see materials and methods section and figure 2) and stored to disk in 24-bit per pixel TIFF format using interactive software developed in C++. Successively they are ingested into the analysis system. The ingestion consists in the extraction of the low-level representative features which permits the retrieval of similar images in terms of colour and texture from the archive. It is important to point out that our approach differs from the ones taken by automatic diagnosis systems aiming to classify the lesion as benign of malignant. Instead of mimicking medical diagnosis methods, such as ABCD rule or other more advanced ones, extracting a multitude of specific parameters, we collect a set of primitive features not related to any specific diagnostic method able to visually characterize the image.

**Figure 3:**
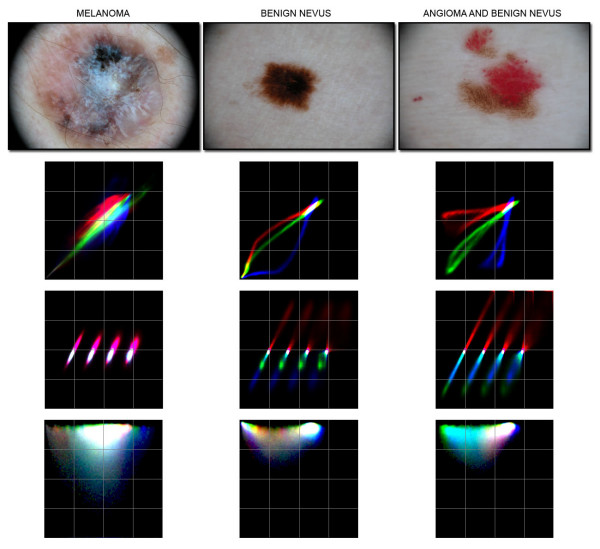
**Representative colour and texture scatter-plots together with the corresponding skin lesion image**. Skin lesion images of a melanoma, a benign nevus and of an angioma combined with a benign nevus and relative colour and texture scatter-plots are shown. The colour scatter-plot is the RGB composition of Red vs. Green, Green vs. Blue and Blue vs. Red channels scatter plots. The co-occurrence matrix is the RGB composition of the three CRCM (Red/Green, Green/Blue and Blue/Red). All CRCM computed at four displacement steps (5, 15, 30 and 60 pixels) are properly shifted in order to avoid interference among the different co-occurrence matrices. The local image variance scatter-plot with respect to the image pixel radiometric value for each band is reported in a RGB composition. The origin of the local variance scatter-plot is located at the upper left corner of the image.

Figure 3 reports representative colour and texture scatter-plots together with the corresponding skin lesion image.

For each feature *ξ *a specific similarity matching function is developed based on a weighted Bhattacharyya distance metric [22]:



where *X *is the normalized scatter-plot feature domain, and *w *is the weighting factor which is specific to each texture feature. The weighting factor *w *is defined in order to minimize the effect of the skin in the similarity matching. In the considered dataset (Caucasian population) the colour scatter-plot of skin is located at high-value grey levels, in the local image variance scatter-plot at low variance value and high grey levels, and in the CRCM at low colour ratio values; therefore *w *decreases moving towards these regions. The downhill Simplex algorithm of Nelder and Mead [23] has been used to obtain the w parameters with a step-size of 0.1. The objective function to maximize is the average retrieval precision score defined in the manuscript.

The proposed distance is completely non-parametric; therefore no assumptions about the feature scatter-plot distribution are made. In order to limit the retrieving response time of the system to few seconds for a set of thousands of images, an efficient hierarchical multi-scale solution has been adopted (an exhaustive explanation of the proposed method is reported in the methods section).

The overall image similarity measure between the query image Q and the database image I is a linear combination of the single similarity matching functions relative to the specific features:



Optimal non negative coefficients  are evaluated experimentally considering the corresponding retrieval quality of the proposed system using the defined PSL archive by 24804 high resolution digital images acquired through the digital dermoscopy system described in the methods section.

Each of the images contained in the archive has been selected as a query image and for each of them the list of the first 20 most similar images together with the relative similitude scores has been stored in a database. These results are statistically analysed computing the average retrieval precision values relative to the group of atypical nevi and to the group of benign ones, where the retrieval precision is the ratio of the relevant retrieved images to the total retrieved ones. A retrieved image belonging to the group of atypical (benign) lesions is considered relevant if the relative query image belongs to the same group of atypical (benign) lesions. Typically the retrieval precision diminishes increasing the number of retrieved images N, because as N is large as it is more probable that visual similar PSLs images belonging to different diagnostic classes are retrieved by the system. This is especially true when the diagnostic class of the query skin lesion is heterogeneous (e.g. Melanoma with atypical pigment network).

In literature it is used also the recall, the ratio of the relevant retrieved images to the total relevant images in the archive [10]. In this specific case the recall parameter is not useful, because the total number of both atypical and benign lesions is orders of magnitude greater than the number of retrieved images.

Therefore, we have used an optimization procedure that computes the precision quality measurement in order to find the best *w *parameters. The best results in terms of average retrieval precision score are given by: . It is evident that the colour similarity matching function is more significant than texture matching functions in retrieving images belonging to the same atypical/benign group, and that within the colour coefficients the blue and red channels are more related to skin lesion pathologies.

Table 2 reports the average retrieval precision values relative to benign nevi, acral nevi and to relevant diagnostic skin lesion categories, which are uniform in the visual appearance by analysing the first N = 5, 10, 20 retrieved images using the optimized similarity matching parameters reported above. It is very important to clarify that the retrieval precision quality measure of a CBIR system is not at all related to the diagnostic accuracy. The reported retrieval precision values show that the proposed CBIR system is actually able to retrieve images visually similar to the query image with respect to colour and texture features and the use of these features is highly effective in finding relevant images for several important diagnostic PSL categories with uniform visual appearance. The system, retrieving visually similar PSLs to the image under evaluation, gives an intuitive aid to clinicians which can improve their diagnostic accuracy.

**Table 2:** Average retrieval precision values by analysing the first N = 5, 10, 20 retrieved images relative to relevant diagnostic skin lesion categories which are uniform in the visual appearance

*Diagnosis*	*Average*	*Retrieval*	*Precision*	*A-priori probability*
	5	10	20	
Melanoma with regression structures	70.0%	52.5%	38.7%	0.11%
Melanoma with atypical pigment network	76.6%	48.3%	35.0%	0.18%
Acral nevi	76.6%	72.5%	69.3%	9.51%
Benign nevi	95.2%	94.4%	94.1%	85.8%
Blue nevi	51.4%	40.0%	30.3%	0.49%
Basal cell carcinomas	40.0%	29.9%	22.2%	0.21%

The percentage of the archive images belonging to each category are reported in order to show the effectiveness of the proposed retrieval system in finding relevant images.

**Figure 4:**
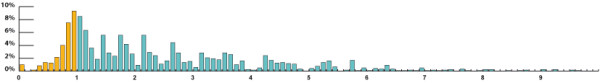
**Frequency distribution of the ratio between average retrieval precision and a-priori probability value relative to the group of atypical nevi**. The analysis has been performed considering the first 12 retrieved images. The average retrieval precision is the ratio of the relevant atypical nevi retrieved images to the total retrieved ones, and the a-priori probability the a-priori probability is the percentage of the atypical nevi with respect to the total number of the archive images.

A similar performance analysis applied to visual heterogeneous diagnostic PSL categories, such as atypical nevi and melanoma is less significant: in same cases only very few PSLs are similar in appearance to the query image. In Figure 4 is reported the frequency distribution of the ratio between average retrieval precision and a-priori probability value relative to the group of atypical nevi, where the a-priori probability is the percentage of the atypical nevi with respect to the total number of the archive images. Considering that a-priori probability of atypical nevi highly overestimates the relevant PSL images a-priori probability contained in the archive, it is evident that the retrieval precision is well above the a-priori probability of the relevant cases. Providing a quantitative assessment is very difficult, because atypical nevi an melanomas cannot be divided in specific homogeneous sub-categories, since the criteria of pattern analysis are multiple and can be present in different combinations in each single lesion [12]. This aspect does not represent a pitfall, since the system is aimed to present to the user the most similar images in the complex of benign, atypical and malignant lesions present in the database respect to the query image. Indeed, the final goal of the system is not to perform a single and definitive diagnosis, but to aid the decision making process of the user.

To note, only CBIR system which model/parameters are defined via intensive training in a supervised way (e.g. Support Vector Machines SVM methods) [24] requires for the system validation a complete different test dataset that are not part of the original database used for the model/parameters definition. In the present case, the proposed system slightly depends on the image dataset of the database. The only dependence is associated in the w parameters (see section "Results") which give the weighting factors for the color, and texture features describing the skin lesion images. These parameters are found using as training set only a subset of 2000 images taken from the whole images database composed by 24804 images (which is very large compared to other databases used in medical CBIR applications).

Finally, we have performed a reproducibility analysis of the proposed CBIR system by selecting as query image a differently scaled and rotated image and evaluating the ranking position that the original image has been given in the retrieved list. A similar analysis has been performed using multi temporal acquisition of the same skin lesion. For rotated and scaled images up to 3× magnification (or 1/3× reduction), the original image is constantly at the firsts position in the retrieved images. For images acquired at very different scale (4-5 relative zoom factors) and for multi temporal PSL acquisitions a few of different images precede the original image in the retrieved ranking list (data not shown).

## Discussion

The proposed system is effective in retrieving PSL images with known pathology visually similar to the image under evaluation giving a valuable and intuitive aid to the clinician in the decision making process. Indeed, we argue that a system, able to retrieve and present cases with known pathology similar in appearance to the lesion under evaluation, may provide an intuitive and effective support to clinicians which potentially can improve their diagnostic accuracy. In addition, this CBIR system can be useful as a training tool for medical students and inexpert practitioners given its ability to browse large collections of PSL images using their visual attributes.

Concerning the possible enhancements of our system with respect to recent state of the art method in computer vision and machine learning such as supervised statistical learning algorithms (for example Support Vector Machines SVM) [24], we would like to point out that the precision results reported in Table 2 is only slightly related to the effectiveness of the tool in giving a support to the clinician in making the diagnosis. The goal of the proposed system is to retrieve PSL images with known pathology visually similar to the image under evaluation. In many cases (especially for the border line PSL) there are several lesions in the archive visually similar to the query lesion but belonging to a different diagnostic category. Showing also these lesions can be useful to the clinician in making the final diagnosis.

The installation of the described system at several medical centres is crucial in order to assess the clinical impact when it is used in clinical practice. The system will allow the user to mark retrieved images as positive and negative relevance feedback. This very important feature will permit both to better evaluate the performance of the proposed system and, consequently, to further tune the weighting factor parameters in order to improve the relevance of the retrieved PSL images. Furthermore, the proposed system can be used to create appropriate CBIR Web Services that can be used remotely to perform query-by-example in various PSL image databases around the world and can be a very good complement to text-based retrieval methods. Finally, a similar search engine finds possible usage in all other sectors of imaging diagnostic, or digital signals (NMR, Video, Radiography, Endoscopy, TAC, etc.), which could be supported by the huge amount of information available in medical archives.

## Conclusion

The system described is able to locate, retrieve and display dermoscopic images similar in appearance to one that is given as a query, using a set of primitive features not related to any specific diagnostic method able to visually characterize the image. Similar search engine could find possible usage in all sectors of diagnostic imaging, or digital signals, which could be supported by the information available in medical archives.

## Competing interests

A.B., R.M. and E.D. are scientific advisers of Advanced Computers System.

## Authors' contributions

AB and LG analyzed the data and wrote the manuscript; RM and ED, together with AB collected all the dermoscopic images; MM, OG and SB defined the mathematical approach and the software described.
